# Pathways affected by asbestos exposure in normal and tumour tissue of lung cancer patients

**DOI:** 10.1186/1755-8794-1-55

**Published:** 2008-11-11

**Authors:** Salla Ruosaari, Tuija Hienonen-Kempas, Anne Puustinen, Virinder K Sarhadi, Jaakko Hollmén, Sakari Knuutila, Juha Saharinen, Harriet Wikman, Sisko Anttila

**Affiliations:** 1 Biological Mechanisms and Prevention of Work-related Diseases, Health and Work Ability, Finnish Institute of Occupational Health, Topeliuksenkatu 41aA, FI-00250 Helsinki, Finland; 2 Department of Information and Computer Science, Helsinki University of Technology, Konemiehentie, Espoo, Finland; 3 Unit of Excellence for Immunotoxicology, Health and Work Ability, Finnish Institute of Occupational Health, Topeliuksenkatu, Helsinki, Finland; 4 Department of Pathology, Haartman Institute, University of Helsinki, and HUSLAB, Helsinki University Central Hospital, Haartmaninkatu, Helsinki, Finland; 5 Department of Molecular Medicine, National Public Health Institute, Mannerheimintie, Helsinki, Finland; 6 Biomedicum Bioinformatics Unit, Biomedicum, Haartmaninkatu, Helsinki, Finland; 7 Department of Tumor Biology, University Medical Center Hamburg-Eppendorf, Martinistrasse, Hamburg, Germany

## Abstract

**Background:**

Studies on asbestos-induced tumourigenesis have indicated the role of, e.g., reactive oxygen/nitrogen species, mitochondria, as well as NF-κB and MAPK signalling pathways. The exact molecular mechanisms contributing to asbestos-mediated carcinogenesis are, however, still to be characterized.

**Methods:**

In this study, gene expression data analyses together with gene annotation data from the Gene Ontology (GO) database were utilized to identify pathways that are differentially regulated in lung and tumour tissues between asbestos-exposed and non-exposed lung cancer patients. Differentially regulated pathways were identified from gene expression data from 14 asbestos-exposed and 14 non-exposed lung cancer patients using custom-made software and Iterative Group Analysis (iGA). Western blotting was used to further characterize the findings, specifically to determine the protein levels of UBA1 and UBA7.

**Results:**

Differences between asbestos-related and non-related lung tumours were detected in pathways associated with, e.g., ion transport, NF-κB signalling, DNA repair, as well as spliceosome and nucleosome complexes. A notable fraction of the pathways down-regulated in both normal and tumour tissue of the asbestos-exposed patients were related to protein ubiquitination, a versatile process regulating, for instance, DNA repair, cell cycle, and apoptosis, and thus being also a significant contributor of carcinogenesis. Even though UBA1 or UBA7, the early enzymes involved in protein ubiquitination and ubiquitin-like regulation of target proteins, did not underlie the exposure-related deregulation of ubiquitination, a difference was detected in the UBA1 and UBA7 levels between squamous cell carcinomas and respective normal lung tissue (p = 0.02 and p = 0.01) without regard to exposure status.

**Conclusion:**

Our results indicate alterations in protein ubiquitination related both to cancer type and asbestos. We present for the first time pathway analysis results on asbestos-associated lung cancer, providing important insight into the most relevant targets for future research.

## Background

Asbestos is a natural mineral fibre with physical and chemical properties that have led to its widespread use for various insulation and construction purposes. Asbestos-exposure is associated with malignancies of the lung and pleura, and tobacco smoking and asbestos-exposure are known to have a synergistic effect on lung cancer risk [1]. Although the use of asbestos is nowadays forbidden or under strict control in most developed countries, asbestos-associated malignancies continue to be a major health problem worldwide due to the long latency period and the extensive usage in the past.

Asbestos fibre characteristics such as length and chemical properties contribute to their toxicity. As macrophages attempt to engulf the fibres, reactive oxygen and nitrogen species (ROS/RNS) are produced. The iron content of asbestos further contributes to the production of these potentially damaging species. ROS/RNS account for several types of DNA and chromosomal damage including formation of mutagenic 8-OHdG adducts and DNA double strand breaks, as well as alterations in signal transduction pathways and apoptosis [2-4].

A major part of the research on asbestos-carcinogenesis is based on animal and *in vitro *models. These studies, including ours on human cell lines exposed to asbestos [5], have indicated numerous changes in several key pathways. The most studied signalling cascades induced by asbestos include the MAPK/ERK and NF-κB pathways [6,7]. Activation of the MAPK cascade affects processes such as cell proliferation, apoptosis, differentiation, and inflammation [7,8]. The apoptotic pathways can be further distorted by alterations in the normal mitochondrial functions, such as induction of cytochrome C release and caspase 9 activation, following asbestos-exposure [9]. Asbestos fibres may also directly interact with the cell cycle machinery that could affect normal cell division [10]. In spite of these observations a comprehensive view on the carcinogenic effects of asbestos fibres still remains largely unknown.

We have recently shown that specific gene copy number and gene expression changes can be detected in lung tumours of asbestos-exposed patients [11,12]. Although several differentially expressed genes were revealed, the single gene approaches that were applied were not suitable for identification of deregulated pathways. To gain further insight into the pathways that are differentially regulated in lung tumours of asbestos-exposed and non-exposed patients we have now performed *in silico *pathway analysis. Differences were sought both in the normal and tumour tissue. While a single gene might not show a significant difference according to asbestos-exposure, moderate differences in a number of genes operating in the same pathway could indicate differential regulation of the whole pathway. Therefore, this approach has the potential of producing a wider mechanistic view on the asbestos-related effects.

We observed several down-regulated pathways that were related to protein ubiquitination in both normal and tumour tissues, whereas the GO terms related to ion transport dominated among the up-regulated pathways. Protein ubiquitination was chosen for further investigation owing to its pivotal role in controlling the replicative potential of a cell. Ubiquitin-activating enzyme E1 (UBA1) and ubiquitin-activating enzyme E1-like (UBA7) were analyzed further due to their roles at the early stages of protein ubiquitination and ubiquitin-like modification processes. To our knowledge, this is the first time when primary tissue samples from asbestos-exposed and non-exposed lung cancer patients have been utilized to perform a comprehensive pathway analysis.

## Methods

### Lung cancer patients

Normal and tumour lung tissue samples were obtained from 14 heavily asbestos-exposed and 14 non-exposed Finnish lung cancer patients as described in Nymark *et al*. [11]. The patients were interviewed for the occupational and tobacco smoking history and their informed consent to participate in the study was obtained. A patient was classified as heavily asbestos-exposed based on employment history and lung tissue asbestos fibre count of more than 5 million fibres per gram of dry lung tissue [13]. The asbestos-exposed and non-exposed patient groups were fit to match the following features: sex (all male), smoking status (current or ex-smokers with similar smoking history), as well as grade, stage and histological type of the tumour. The study protocols were approved by the Ethical Review Board for Research in Occupational Health and Safety, Helsinki and Uusimaa Hospital District (75/E2/2001).

### Gene expression and pathway analysis

Gene expression analyses on normal and tumour tissue samples from the 28 lung cancer patients were performed using Affymetrix HG-U133A GeneChips (Affymetrix, Santa Clara, CA, USA) as previously described [12]. After scanning the chips the gene expression data were preprocessed using the GC Robust Multi-array average method [14]. The Gene Ontology (GO) Database [15,16] was searched for pathway (GO term) information for the genes represented on the chip. Annotations to biological processes, molecular functions, and cellular localization were utilized in the analyses. Custom-made software and Iterative Group Analysis (iGA) [17] were used to obtain information on the differentially regulated pathways.

First, the genes were rank ordered with respect to differences in gene expression between the two groups of patients. Both p-values from the t-test and the fold-change between the medians of the two groups were used in performing the ranking, thus two rank lists were obtained. Comparisons between the groups were performed on normal tissue vs. normal tissue and tumour vs. tumour, separately for both up- and down-regulated pathways. iGA was then applied to test the categories defined by GO for enrichment of over or under expressed genes, separately for the t-test and fold-change based rank lists. This approach was chosen due to the complexity of carcinogenic processes in the lung. The complexity makes the identification of the most important differences between the tumour and normal tissues of the asbestos-exposed using genome-wide gene expression difficult. Owing to this inherent difficulty we chose to compare tumour vs. tumour and normal vs. normal and integrate the results to decipher changes specifically caused by exposure to asbestos.

iGA was applied on the ranked lists to test each pathway for enrichment of differentially expressed genes. The method does not require defining of thresholds for differential expression but instead determines an optimal p-value for each pathway by considering all possible options for the number of over- or under-expressed genes. The final permuted p-values were obtained by means of hypothesis testing using 50,000 permutations of the group labels.

### Western blot

To determine the protein expression levels of UBA1 and UBA7, we performed Western blotting on fresh frozen tumour and respective normal lung tissue from asbestos-exposed and non-exposed lung cancer patients. A total of six asbestos-exposed and six non-exposed cases were included in the analyses with both groups containing three adenocarcinomas (ACs) and three squamous cell carcinomas (SCCs). Cytoplasmic and nuclear proteins were extracted from fresh frozen tissue samples using NE-PER extraction kit according to the protocol provided (Pierce, Rockford, IL). The percentage of tumour tissue on frozen sections from the same samples was evaluated by a pathologist before protein extraction and was typically around 50%. Protein concentration was measured using DC protein assay (Bio-Rad, Hercules, CA) and equal amounts of proteins of the cytoplasmic fraction were loaded onto 10–20% Criterion Tris-HCl gels (Bio-Rad). Proteins were blotted onto Immobilon-P PVDF membrane (Millipore, Billerica, MA, USA). UBA1 and UBA7 detections were performed using the following antibodies: rabbit polyclonal antibodies to UBA1 (ab16849) and UBA7 (ab12199) (Abcam, Cambridge, UK) at 1:5000 and 1:2000 dilutions, respectively. Rabbit polyclonal antibody to GAPDH (ab9485, Abcam) at a 1:2000 dilution was utilized as an internal control. Biotinylated anti-rabbit IgG (Vector Laboratories Inc., Burlingame, CA, USA) at a 1:15000 dilution together with streptavidin-horseradish peroxidase conjugate (Amersham Biosciences, NJ, USA) at a 1:5000 dilution were applied. The signal was detected by using an ECL reagent (Amersham Biosciences), and the UBA1, UBA7, and GAPDH band intensities were quantified with an ImageMaster program (Amersham Biosciences).

## Results

### Asbestos-related pathways

**Figure 1:**
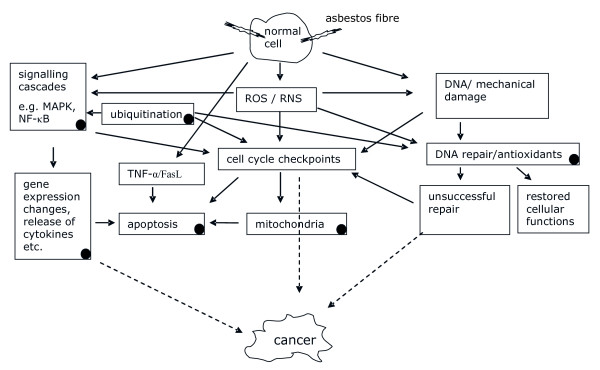
**Presentation of asbestos-induced effects**. Schematic presentation of asbestos-induced effects, modified from Upadhyay and Kamp [38]. Mechanisms or cellular compartments affected by asbestos-exposure according to the pathway analysis are marked with black dots.

When GO terms from all levels of specificity were considered, several differentially regulated pathways (GO terms) were identified both in normal and tumour tissue of asbestos-exposed patients as compared to the non-exposed (Additional files 1 and 2). The detection of a large number of potentially affected pathways was expected owing to the presence of thousands of GO terms, all of which were tested for differences between the two groups. The findings implicated alterations in many vital aspects of cell homeostasis (Figure 1). The pathway analysis revealed some previously presented asbestos-associated molecular alterations, such as deregulation of NF-κB pathway that was observed in the normal tissue samples from asbestos-exposed patients. Additional somewhat expected findings included changes in DNA repair and mitochondrial functions.

**Figure 2:**
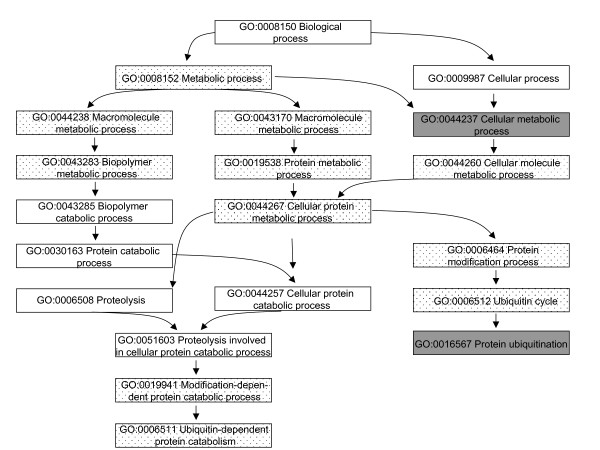
**Protein ubiquitination related differentially regulated processes**. The dotted boxes represent GO terms that were down-regulated in both the normal and the tumour tissues of the exposed patients in comparison to the non-exposed using both the t-test and fold change based ranking. The grey boxes represent GO terms that were down-regulated using at least one of the tests.

**Figure 3:**
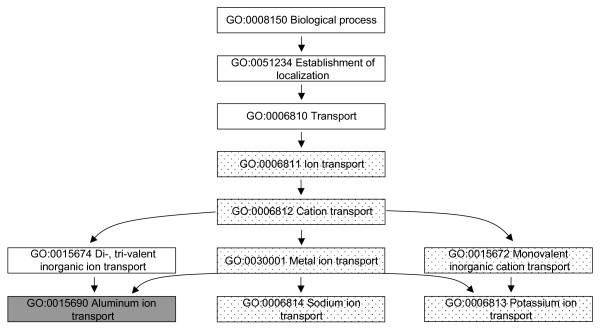
**Ion transport related differentially regulated processes**. The dotted boxes represent GO terms that were up-regulated in both the normal and the tumour tissues of the exposed patients in comparison to the non-exposed using both the t-test and fold change based ranking. The grey boxes represent GO terms that were up-regulated using at least one of the tests.

The GO terms share a parent-child relationship in which the children are detailed descriptions of the parents. Hence, the terms can be organized into a treelike structure where each branch contains related cellular functions, processes or locations. To pinpoint perhaps the most relevant findings in our data we sought for those branches that contained at least three GO terms with permuted p-values < 0.05 obtained using both t-test and fold change based ranking. Furthermore, we were interested in identifying categories from which targets for additional analyses could be selected and thus considered only branches with significant results on categories containing less than 100 genes. Consequently, we identified 30 down-regulated and 40 up-regulated pathways in the normal tissue, and 22 down-regulated and 37 up-regulated pathways in the tumour tissue (data not shown). 27 up-regulated and 8 down-regulated pathways detected in the tumour were in common with those in the normal tissue (Additional file 3). Figures 2 and 3 shows the differentially regulated biological processes from all levels of specificity related to one of the up-regulated and one of the down-regulated processes, ion transport and protein ubiquitination, respectively.

Among the most intriguing findings were the up-regulated pathways associated with ion transport and the down-regulated pathways associated with protein ubiquitination both in the normal and tumour tissues. Six out of 24 biological processes and molecular functions up-regulated in both normal and tumour tissues of the exposed patients were ion transport related, whereas three out of the eight down-regulated terms were related to ubiquitination (see Additional file 3). Interestingly, our previous investigations of time-dependent gene expression changes of three cell lines (A549, BEAS 2B and Met5A) also indicated modifications in protein ubiquitination and ion transport after exposure to crocidolite asbestos fibres [5]. In that study, we performed similar pathway analysis as in the current work and found deregulation of ubiquitination related GO terms in all three cell lines 24 h or 48 h after the exposure [5]. Coinciding findings from patient samples and *in vitro *models could imply importance of ubiquitination in asbestos-related lung carcinogenesis.

### UBA1 and UBA7 in lung tumours and normal lung

To assess the potential importance of UBA1 and/or UBA7 in asbestos-associated lung cancer or lung carcinomas in general we measured the protein levels in normal and tumour tissue samples of 12 lung cancer patients (data not shown). Both exposed and non-exposed study groups contained three ACs and three SCCs and the corresponding normal tissue samples.

The GAPDH-scaled protein expression levels of UBA1 and UBA7 were compared between the exposed and non-exposed lung cancer patients, separately for the normal and tumour tissue samples. The expression levels were also compared between the normal and tumour samples, separately for AC and SCC cases, irrespective of the exposure status of the patients. We did not detect exposure-related differences in UBA1 or UBA7 levels in the normal or tumour tissue samples. In the SCC group, however, protein expression of both UBA1 and UBA7 were significantly lower in tumour than in the normal tissue, p = 0.02 and p = 0.01 (two-sided t-test), respectively. The results are summarized in Table 1. The gene expression microarray data also showed that *UBA1 *and *UBA7 *mRNA levels were significantly lower in the SCCs compared to the respective normal tissue samples (Table 1).

**Table 1:** The protein expression levels of UBA1 and UBA7 in the tumour and normal tissue of adenocarcinoma and squamous cell carcinoma patients obtained using Western analysis and gene expression (GE) microarrays

	UBA1		UBA7		
	Western	GE probe 1	Western	GE probe 1	GE probe 2
Tissue type^a^	Median [range]		Median [range]		
SCC	0.72 [0.29 0.91]	1097 [827 1410]	0.16 [0.13 0.27]	71 [54 88]	84 [67 95]
Normal	1.3 [0.81 2.8]	1723 [1037 1815]	0.74 [0.50 1.7]	131 [108 170]	194 [147 253]
p-value^b^	0.02	0.03	0.01	0.00086	0.00018
AC	1.1 [0.70 1.2]	1326 [658 1656]	0.50 [0.15 0.62]	104 [72 160]	122 [68 228]
Normal	1.1 [0.24 1.9]	1162 [932 1601]	0.46 [0.11 0.95]	137 [119 221]	203 [162 406]
p-value^b^	0.80	0.42	0.79	0.01	0.003

^a^AC, adenocarcinoma; SCC, squamous cell carcinoma

^b^p-value was calculated using two-sided t-test

## Discussion

Here we have presented, for the first time, comprehensive pathway analysis on asbestos-related lung cancer. Fresh frozen tissue samples were analyzed from well-defined patient material consisting of asbestos-exposed and non-exposed lung cancer patients. Gene expression measurements together with gene annotation data from the Gene Ontology database were utilized to identify potential asbestos-associated pathways.

The pathway analysis provides an attractive means for detecting gene expression changes that have occurred in a number of genes involved in the same biological processes or molecular functions, or located in the same cellular compartment. At the gene level, the changes in expression do not have to be significant, opposed to the single gene approaches that are commonly used to analyze high-throughput data. Moreover, as the samples in both the exposed and non-exposed groups represented different tumour types, the analysis was insensitive to the differences between the histological tumour types.

We observed a multitude of differences between the asbestos-associated and non-associated lung cancer in several key cellular events. In order to pinpoint the truly meaningful molecular changes and to reduce the chance of false findings resulting from the large number of statistical tests that are being made the significance thresholds should be stringent enough. To pinpoint the most relevant findings in our data, we performed a two-step analysis, where the significance of each category was first assessed by enrichment of differentially expressed genes in the category. The significance of the categories was evaluated by means of hypothesis testing using 50,000 permutations of the group labels. Both p-values from the t-test and fold-change between the group medians were used as measures of differential expression. Thereafter, we required that at least three components of a GO branch had to be significant according to the two measures. In addition to the challenges related to performing multiple statistical tests, it is noteworthy that the pathway analysis is based on the current knowledge of gene functions and processes occurring in the cellular environment, which has been derived using versatile methods. Therefore, future findings may unravel further differences between the asbestos-exposed and non-exposed or provide deeper insight into the differences between these two groups.

Some of the characteristics observed in the asbestos-exposed group have already been presented in the literature, indicating the utility of this approach. Interestingly, ion channel related GO terms dominated the list of up-regulated pathways. Asbestos could be linked to up-regulation of ion channels through ROS, which has been shown to trigger the opening of mitochondrial channels and mitochondrial depolarization [18]. Ion channel defects have been observed to underlie various diseases [19-21], but also suggested to have a significant role in tumourigenesis and tumour progression [[22], reviewed in [23]]. Ion channels could be an important target for further studies, also in relation to asbestos, because they contribute to virtually all basic cellular processes such as proliferation, differentiation, and apoptosis [[24], reviewed in [25]].

Among the down-regulated pathways, biological processes and molecular functions related to protein ubiquitination, a versatile process regulating various key cellular events such as DNA repair, cell cycle, and apoptosis [26], were the most intriguing findings. Furthermore, many of the other significantly altered pathways identified here are connected to ubiquitin-mediated cellular trafficking and degradation by the proteasome. These include, e.g., clathrin-coated vesicles and modification-dependent protein catabolism as well as pathways implicated in transport between Golgi and endoplasmic reticulum. Also, asbestos could have a direct interaction with cellular constituents [7] such as the nucleosome and spliceosome complexes. Of note, deregulated ubiquitination has been linked to carcinogenesis [27], especially to mesothelioma [[28,29] (unpublished data)], a disease tightly linked to asbestos-exposure.

We chose protein ubiquitination as a target for further analysis due to its versatile role in many aspects of cellular homeostasis and, e.g., the replicative potential of a cell and as it therefore may in fact underlie several of the previously presented molecular alterations in asbestos-associated carcinogenesis. Due to limited availability of ample fresh frozen samples we chose two targets for further research. Namely, the protein levels of UBA1 and UBA7 in the tumour and respective normal tissue of asbestos-exposed and non-exposed patients were analyzed due to their role at the early stages of protein ubiquitination and ubiquitin-like modification processes. UBA1, an E1 enzyme, initiates the addition of ubiquitin signalling tags to target proteins. E1 enzyme transfers ubiquitin to E2 enzyme, which together with E3 enzyme subsequently tags the substrate [26]. Proteins involved in ubiquitin-like modification processes, such as UBA7, seem also to play a role in the ubiquitin-proteasome system [30,31]. UBA7 strikes as an interesting candidate also because of the low expression levels detected in lung cancer cell lines and the location of the gene on 3p21, a region frequently deleted in lung carcinomas associated with tobacco smoking and asbestos-exposure [11,32,33].

According to our analysis of a rather small number of normal and tumour tissue samples the asbestos-related deregulation of ubiquitination seems neither to be caused by UBA1 nor UBA7. However, several other enzymes are also involved in protein ubiquitination besides UBA1 and UBA7. To identify the asbestos-associated alterations of the pathway, more holistic approaches analyzing a multitude of associated proteins in a large number of samples could be useful. Mass spectrometry analyses or immunohistochemistry performed on tissue arrays, applied on normal and tumour tissues of asbestos-exposed and non-exposed lung cancer patients, might reinforce the data presented here. Furthermore, imaging techniques on cell lines could provide additional information on the possible deregulations in ubiquitination.

We observed under-expression of UBA1 and UBA7 in SCCs, but not in ACs, both at the mRNA and protein level. This finding appears to contradict some studies, which have suggested a higher risk of AC than SCC in association with asbestos-exposure [34]. Several epidemiological studies on asbestos-related cancer have shown, however, similar risk ratios for all major histological types of lung cancer [35-37]. The results thus indicate both asbestos and cancer type specific alterations in protein ubiquitination with different components of ubiquitination pathway potentially contributing to different types of lung cancer. Future work is needed to understand the alterations connected with asbestos and histological cancer types

## Conclusion

The pathway analysis depicts a thorough picture of the molecular changes induced by asbestos-exposure and indicates that there are distinct differences between asbestos-related and non-related lung tumourigenesis. Specifically, up-regulation of the pathways associated with ion transport and down-regulation of the functions related to protein ubiquitination were found in the normal and tumour tissue of exposed patients. The data will provide the research community important insight into the relevant targets underlying asbestos-associated lung cancer.

## Abbreviations

GO: Gene Ontology; UBA1: ubiquitin-like modifier activating enzyme 1; UBA7: ubiquitin-like modifier activating enzyme 7; ROS/RNS: reactive oxygen/nitrogen species; AC: adenocarcinoma; SCC: squamous cell carcinoma; iGA: iterative group analysis.

## Competing interests

The authors declare that they have no competing interests.

## Authors' contributions

SR identified the most significant GO branches, performed the statistical analyses apart from pathway analyses and contributed to the Western blot analyses and writing of the manuscript. TH-K analyzed the pathway data with respect to current literature, drafted the manuscript and performed Western blot analyses. AP oversaw the protein analysis work and helped revise the manuscript. VKS participated in performing microarray analyses and contributed to the scientific discussions of the project. SK supervised the microarray analyses. JH contributed to statistical analyses and together with SK contributed to the scientific discussions of the project. JS performed the pathway analyses. HW performed the microarray analyses, initiated the pathway analysis project and revised the manuscript. SA was responsible for the collection of human samples and overall study design and oversaw the project. All authors read and approved the manuscript.

## Pre-publication history

The pre-publication history for this paper can be accessed here:



## Supplementary Material

Additional file 1 **Up-regulated pathways in the normal (N) and tumour (T) tissue identified using the t-test and fold change (fc)**. The results are presented for the terms for which at least one of the four tests gives p < 0.001 (shown in italics). P-values > 0.2 are given as na. Click here for file

Additional file 2 **Down-regulated pathways in the normal (N) and tumour (T) tissue identified using the t-test and fold change (fc).** The results are presented for the terms for which at least one of the four tests gives p < 0.001 (shown in italics). P-values > 0.2 are given as na. Click here for file

Additional file 3 **Pathways that were differentially regulated both in the normal and tumour tissue of asbestos-exposed patients compared to non-exposed patients.** Only the most specific GO terms are presented as the terms share a parent-child relationship where children are detailed descriptions of the parents. The presented GO terms were found to be differentially expressed using both t-test and fold change based ranking with permuted p-value < 0.05. Click here for file
