# Supplementary material for: Pathways affected by asbestos exposure in normal and tumour tissue of lung cancer patients
Source: BMC Med Genomics. 2008 Nov 11;1:55. doi: 10.1186/1755-8794-1-55 (PMC2612681; doi:10.1186/1755-8794-1-55)
Supplement: Additional file 3 — Pathways that were differentially regulated both in the normal and tumour tissue of asbestos-exposed patients compared to non-exposed patients. Only the most specific GO terms are presented as the terms share a parent-child relationship where children are detailed descriptions of the parents. The presented GO terms were found to be differentially expressed using both t-test and fold change based ranking with permuted p-value < 0.05. [file 1755-8794-1-55-S3.doc]

Biological processes U/D^a^

GO:0006813 potassium ion transport U

GO:0006814 sodium ion transport U

GO:0006816 calcium ion transport U

GO:0006875 metal ion homeostasis U

GO:0007188 G-protein signaling, coupled to cAMP nucleotide 2nd m. U

GO:0007200 G-protein signaling, coupled to IP3 second messenger U

GO:0007214 gamma-aminobutyric acid signaling pathway U

GO:0007215 glutamate signaling pathway U

GO:0007223 frizzled-2 signaling pathway U

GO:0007601 visual perception U

GO:0007608 perception of smell U

GO:0007631 feeding behavior U

GO:0019735 antimicrobial humoral response (sensu Vertebrata) U

GO:0030005 di-, tri-valent inorganic cation homeostasis U

GO:0042095 interferon-gamma biosynthesis U

GO:0006399 tRNA metabolism D

GO:0006511 ubiquitin-dependent protein catabolism D

GO:0043037 translation D

Molecular functions

GO:0004890 GABA-A receptor activity U

GO:0004984 olfactory receptor activity U

GO:0005184 neuropeptide hormone activity U

GO:0005231 excitatory extracellular ligand-gated ion channel activity U

GO:0005249 voltage-gated potassium channel activity U

GO:0005262 calcium channel activity U

GO:0008009 chemokine activity U

GO:0008188 neuropeptide receptor activity U

GO:0008227 amine receptor activity U

GO:0004839 ubiquitin activating enzyme activity D

GO:0008026 ATP-dependent helicase activity D

GO:0008642 ubiquitin-like activating enzyme activity D

GO:0016886 ligase activity, forming phosphoric ester bonds D

GO:0046966 thyroid hormone receptor binding D

Cellular compartments

GO:0005882 intermediate filament U

GO:0008076 voltage-gated potassium channel complex U

GO:0045211 postsynaptic membrane U

^a^U, upregulated in asbestos-exposed patients compared to non-exposed patients; D, downregulated in asbestos-exposed patients compared to non-exposed patients
